# Highly Efficient and Specific Genome Editing in Silkworm Using Custom TALENs

**DOI:** 10.1371/journal.pone.0045035

**Published:** 2012-09-18

**Authors:** Sanyuan Ma, Shengling Zhang, Feng Wang, Yong Liu, Yuanyuan Liu, Hanfu Xu, Chun Liu, Ying Lin, Ping Zhao, Qingyou Xia

**Affiliations:** State Key Laboratory of Silkworm Genome Biology, Southwest University, Chongqing, China; National University of Singapore, Singapore

## Abstract

Establishment of efficient genome editing tools is essential for fundamental research, genetic engineering, and gene therapy. Successful construction and application of transcription activator-like effector nucleases (TALENs) in several organisms herald an exciting new era for genome editing. We describe the production of two active TALENs and their successful application in the targeted mutagenesis of silkworm, *Bombyx mori*, whose genetic manipulation methods are parallel to those of *Drosophila* and other insects. We will also show that the simultaneous expression of two pairs of TALENs generates heritable large chromosomal deletion. Our results demonstrate that (i) TALENs can be used in silkworm and (ii) heritable large chromosomal deletions can be induced by two pairs of TALENs in whole organisms. The generation and the high frequency of TALENs-induced targeted mutagenesis in silkworm will promote the genetic modification of silkworm and other insect species.

## Introduction

Precise genome editing is crucial for functional genomic research and exploratory research. Although gene targeting has been used in some model organisms for more than 20 years [1], genome editing has never been easy for non-model organisms. Therefore, establishing an efficient and precise genome editing method for both model and non-model organisms is urgent, especially for economically or ecologically important organisms. Zinc finger nucleases (ZFNs) are chimeric proteins engineered to facilitate genome editing by introducing a double-stranded break (DSB) at specified locations [2]. ZFNs have become the most powerful tools for the genomic manipulation of many plants and animals and various types of mammalian cells in basic research, agriculture, and therapeutic applications [3]. However, the generation of custom ZFNs that target a desired sequence with high specificity and activity remains a challenge, mainly due to the lack of known fingers for some nucleotide triplets and the context effects of individual fingers in an array [4]. TAL effector nucleases (TALENs), recently discovered tools, are similar in function to ZFNs. Instead of the zinc finger motif in ZFNs, the DNA binding motif of a TALEN is an engineered transcription activator-like effector (TAL effector) protein. TAL effectors recognize DNA in a modular fashion: two hypervariable amino acid residues in each tandemly arrayed repeat independently recognize single, contiguous nucleotides in the DNA target [4], [5]. Compared with ZFNs, TALENs are much more predictable and simple for researchers to edit the genome at will. Since Christian et al. [6] reported their first attempt to design custom TALENs for genome editing in cultured human cells and Zhang et al. [7] developed a strategy to construct sequence-specific TAL effectors; applications for TALEN have been reported in a number of organisms, including yeast [8], roundworms [9], rats [10], zebrafish [11], [12], Arabidopsis protoplasts [13], human embryonic stem cells (ESCs), and induced pluripotent stem cells (IPSCs) [14].

TALENs have not yet been demonstrated to induce (i) targeted disruptions in silkworm and (ii) sophisticated genetic modifications other than disruptions in organisms. Genetic manipulations are powerful tools that enable the basic study of model insects, exploratory studies on insects, and the control of insect vectors. Although the ability to generate genetically modified insects has become a reality in some key model systems over the past 15 years [15], most of the methods were based on transposon vectors. Increasing scientific and public concerns were raised toward transposon vector derived genetically modified organisms, mainly because of limitations such as random insertion, low transformation frequency, possible instability, and limited carrying capacity [15]. Despite continuous efforts, such as the improvement of transposon vector systems [16], [17], the utilization of site specific recombinases [18], and the establishment of gene targeting strategies [19], [20], as well as a few applications of ZFNs in *Drosophila* [21]–[23] and in silkworms [24], genome editing remains a great challenge in most of insect species. The use of TALENs may overcome the limitations of current genetic modification technologies. In many cases of genome manipulation, deletions of several hundred base pairs or longer are expected. Two strategies using two ZFNs [25], [26] and ZFNs together with a single stranded oligodeoxynucleotide (ssODN) [27] were proven to enable targeted chromosomal deletions in human cell lines. Given the simplicity and efficiency of TALENs, sophisticated genome manipulation such as using TALENs to induce large deletions may provide new possibilities for the genetic manipulation of insect species and, likely, other eukaryotic systems.

The study explores the utility of TALENs for genome editing in the silkworm, *Bombyx mori*, whose genetic manipulation methods parallel those of *Drosophila* and other insects. Two TALENs that target the 2^nd^ and 3^rd^ exon of *BmBlos2*, which is responsible for the transparent skin phenotype [24], were generated using direct synthesis. Microinjection of both TALENs induced high frequency mosaic and germline mutations. Moreover, the heritability of the large chromosomal deletions through simultaneous expression of two pairs of TALENs was demonstrated.

## Materials and Methods

### Design and Construction of TALENs

The TALENs were designed using 240 N-terminal and 63 C-terminal truncations according to Zhang *et al* [7]. The RVD (repeat variable di-residue) of each repeat was designed following the general rule of TALEN recognition (NG, HD, NI, and NN recognize T, C, A, and G, respectively) with six exceptions. Considering that TAL effectors have more types of RVD, six non-canonical RVDs were used in the design. These changes were based on the occurrences of RVD at corresponding locations within the repeat in natural TAL effectors [28]. Two additional amino acids were added to both the N- and C-terminals to form two enzyme cleavage sites. The designed amino acid sequences of TALENs (designated as B2L, B2R, B3L, and B3R) were converted to DNA sequences that correspond with the codon usage in silkworm and then synthesized using a commercial service (GenScript). Two vectors containing T7 promoters with an NEL or CKK *Fok*I variant were provided by Sigma-Aldrich. B2L/B3L and B2R/B3R DNAs were cloned into the NEL and CKK *Fok*I vectors, respectively, using *Kpn*I + *Bam*HI digestion. All the vectors constructed were sequenced using primers flanking the entire coding regions to confirm the DNA synthesis and cloning (Text S1). All the methods used for vector construction followed the standard molecular cloning protocols and instructions provided in the kits.

### mRNA Synthesis

Plasmid DNA was prepared using a plasmid midi kit (QiaGen), digested with *Xba*I (Promega) and phenol/chloroform purified. mRNA of B2L, B2R, B3L, and B3R were transcribed and polyadenylated *in vitro* using the MessageMax™ T7 ARCA-capped Message Transcription Kit and Poly (A) Polymerase Tailing Kit (Epicentre Biotechnologies). The resulting mRNA was purified using the MegaClear Kit (Ambion) before resuspension in RNAse-free water (Sigma-Aldrich) and then quantified using a NanoDrop-1000 (Thermo Scientific). B2L and B3L were mixed with B2R and B3R at a molar ratio of 1∶1 and stored at −80°C until use.

### Microinjection and Analysis of Mosaic Mutations

Nistari, a non-diapausing silkworm strain, reared on fresh mulberry leaves, was used in the study. Embryos were prepared for microinjection as described by Tamura et al. [29] and Zhao et al. [30]. B2 mRNA was dissolved at 700 ng/µl and B3 mRNA was dissolved at 700, 400, and 200 ng/µl. The mRNA samples were microinjected into the silkworm embryos within 2 h after oviposition. The microinjections were performed using TransferMan NK2 micromanipulator and Femto Jet 5247 microinjector (Eppendorf) under an SZX16 microscope (Olympus). The injection opening was sealed with instant glue (Konishi Co.), and the injected embryos were incubated at 25°C and 90% relative humidity. The larvae that hatched from the injected embryos were collected and reared on fresh mulberry leaves. Considering the microinjection delayed the hatching of some silkworms in every injection group, the larvae that hatched at different days were reared separately. Mosaic mutations were observed as early as the 3rd instar. The statistical data were collected on the 5^th^ day of the 5^th^ instar, at which the phenotype of some mosaic mutants, those with mostly normal (opaque) integument and small translucent areas, could be observed.

### Crossing Strategy and Creation of Germline Mutations

Silkworm females are hemizygous for the Z chromosome where the BmBlos2 gene is located, thus indicating that mutations occurring in male germ cells will generate detectable mutations in the next generation of females. During strain maintenance, G0 males were first crossed with available G0 females; the remaining unpaired G0 males were crossed with wild-type females. Using this strategy, all the mutations from the G0 males and some of the mutations from the G0 females were tested. The G1 progeny were checked for the translucent skin phenotype at the 3rd instar, and all positive mutants were allowed to develop to the 5th instar to determine gender. Some of the mutants were also allowed to develop to the pupal stage to confirm gender.

### Sequencing of the Mutations

For the G1 mutations, only the females were subjected to DNA extraction and sequencing. The genomic DNA was extracted using nucleic acid isolation systems PI-1200 (Kurabo), and the PCR amplified segments were sequenced directly using a 3730 DNA analyzer (ABI). The methods used for PCR and sequencing followed standard molecular cloning protocols and the instructions provided by the manufacturers.

## Results

### Design of TALENs and Construction of Expression Vectors

**Figure 1:**
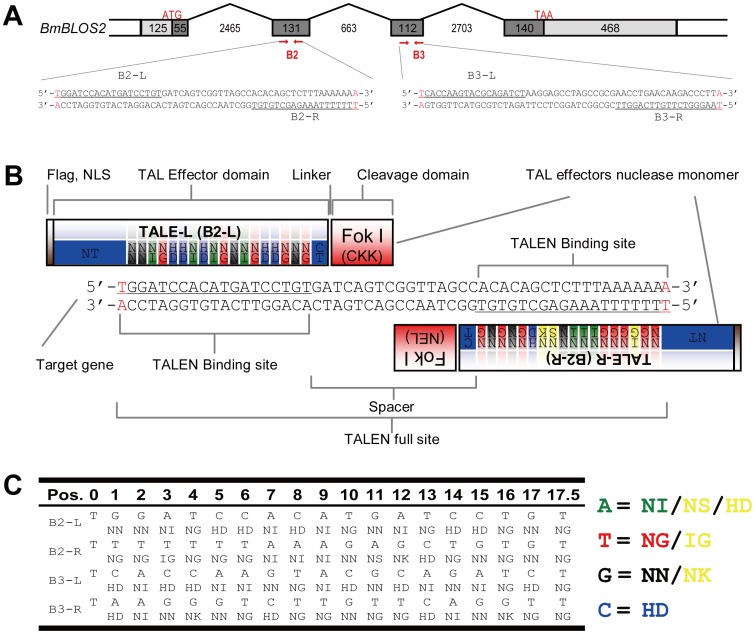
Design and construction of TALENs that target the *BmBlos2* gene. (A) Schematic representation of the *BmBlos2* gene structure depicting the introns (broken lines), exons (colored boxes) and TALEN target sequences (sequences at the bottom). The numbers indicate the exact lengths of the introns and exons. ATG and TAA indicate the translation start and stop sites, respectively. The underlined sequence represents the recognition site of the corresponding TALEN monomer. (B) Schematic representation of TALEN binding to its target DNA. A triple flag tag and a nuclear localization signal (NLS) (grey boxes) are fused to the N-terminal of each TALEN monomer. The *Fok*I domain (red boxes) is linked to the C-terminal of each TALEN monomer through a flexible linker. The TAL effector domain is composed of an N-terminal (blue box labelled NT), a C-terminal (blue box labelled CT), and a tandem array of repeats (an array of colored boxes between NT and CT). The TALEN binding site is composed of a left binding site (underlined), right binding site (underlined) and a spacer (NNN…). (C) TALEN target sequences and the corresponding RVDs within each repeat. The number at the top of the left panel indicates the position of the repeat. The letters at the top of each TALEN monomer represent the target sequence and the letters below represent the RVDs of the corresponding repeat. The right panel represents the RVD usage in the design. The yellow coloured RVDs are different from the commonly used RVDs.

To avoid effects such as chromatin structure and DNA methylation, and to directly compare TALENs with ZFNs, *BmBlos2*, the only gene successfully edited by Takasu et al. [24] using ZFNs in silkworm, was selected as the target gene. *BmBlos2* is located on the Z chromosome and is responsible for the transparent skin phenotype [24]. Both of these characters simplify screening. Two TALENs, B2 and B3, were designed to target the 2^nd^ and 3^rd^ exons, respectively (Figure 1A and B). Neither the B2 nor the B3 target site has homologous sequences in the genome. NG, HD, NI, and NN RVD were considered to recognize T, C, A, and G, respectively; however, TAL effectors have more types of RVDs despite lower occurrence. Therefore, six non-canonical RVDs were used in the design at locations that correspond with the natural TAL effectors (Figure 1C). DNA sequences that encode the TAL effector domains of the four TALEN monomers were synthesized. To facilitate the synthesis of multiple directional repeated sequences, the codons were optimized according to the codon usage in silkworm. The synthesized fragments were inserted into expression vectors containing the T7 promoter and *Fok*I coding sequence (Sigma-Aldrich). To avoid the formation of homodimers, two variant *Fok*I (Sigma-Aldrich) were used (Text S1).

### High Frequency TALEN-induced Mosaic Mutagenesis

**Figure 2:**
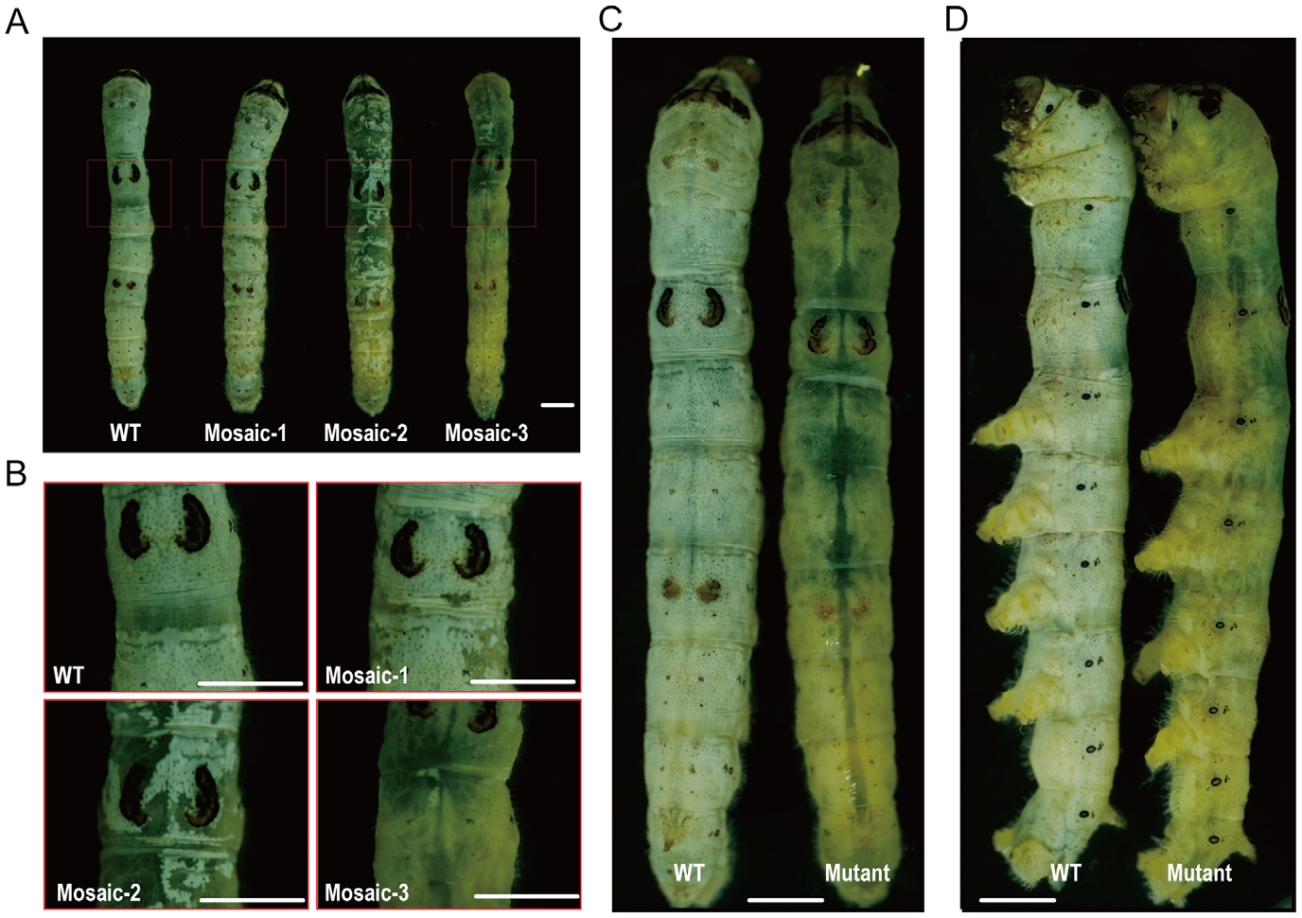
Phenotype of mosaic mutations and germline mutations. (A) Wild type (Nistari) and G0 individuals with mosaic mutations. Mosaic-1, Mosaic-2, and Mosaic-3 represent the three different types of mosaic mutations observed. (B) Mosaic mutation (A) magnified. The epidermal cells were completely opaque in the wild-type silkworms, mostly opaque with translucent spots in G0 Mosaic-1 mutant silkworms, half opaque and half translucent in G0 Mosaic-2 mutant silkworms, and mostly translucent with opaque spots in G0 Mosaic-3 silkworms. (C) Germline mutations from the dorsal side. (D) Germline mutations from the lateral side. The epidermal tissue was completely translucent in the germline mutations. The scale bar represents 5 mm.

The *in vitro*–synthesized mRNA was introduced into the silkworm embryos through microinjection. Up to 66/144 (46%) of the G0 silkworms injected with B2 mRNA and 28/126 (22%) of the G0 silkworms injected with B3 mRNA showed mosaic mutations at the 5^th^ instar (Figure 2A, B, and Table S1). The translucent integument did not preferentially manifest on the ventral side or dorsal side, which differs from the phenomenon observed by Takasu et al. [24]. We suspect that this difference may be caused by the position of injection on the embryos. The frequency of mosaic mutations was dose-dependent, with the highest percentage of mosaic mutations (27%) occurring in the insects injected with 700 ng/µl mRNA (Figure S1B); however, even at 200 ng/µl, the percentage was as high as 10%, comparable to ZFN-induced mutations in silkworm (0%–72%) [24]. The difference in frequency of mosaic mutation between the two TALENs at the same dose was probably due to the difference in activity of the single custom designed TALENs (Figure S1A). As observed previously, microinjection can lead to the delayed hatching of some embryos. The hatched silkworms were collected daily and reared separately. Interestingly, we observed a much higher frequency of mosaic mutations in the delayed hatched silkworms (Figure S1C). To test whether the mutations occurred in a single allele (female) or two alleles (male), the mosaic silkworms were observed at the pupal stage when the sexes can be accurately distinguished [24]. Interestingly, 12/26 (46%) of the B2 mRNA–induced mutants and 7/9 (78%) of the B3 mRNA–induced mutants were males, which suggests that both TALENs could induce high frequencies of biallelic mutations (Table S1).

### Efficient Germinal Transmission of TALEN-induced Mutations

To determine whether these mutations were heritable, we bred G1 broods from the injected silkworms through sibling crossing or backcrossing. Considering the possibility of obtaining mutations from the non-mosaic G0 silkworms, both the mosaic and non-mosaic silkworms were mated. The G1 eggs were allowed to hatch and the larvae were reared by brood to the 3^rd^ instar to screen for mutations. Both TALENs induced a much higher frequency of heritable mutations than ZFN [24]. 9/29 (31%) broods of B2 and 6/41 (15%) broods of B3 contained mutant individuals ranging from 1 (0.4%) to 176 (61%) per brood (Figure 2C, D, and Table S1). Of all broods containing mutant silkworms, 14/21 (67%) were from mosaic silkworms and 3/63 (5%) were from non-mosaic silkworms (Figure S1D), which indicates that the non-mosaic silkworms also yielded germline mutations, but at a low frequency. A total of 630 mutant G1 silkworms were obtained from the B2 mRNA injected silkworms, whereas 277 were from those injected with B3 mRNA. 104 B2 and 85 B3 mutant silkworms were allowed to develop to the pupal stage, among which 21 (20%) and 7 (8%) were male, respectively (Table S1).

### Determination and Analysis of the Mutant Sequences

**Figure 3:**
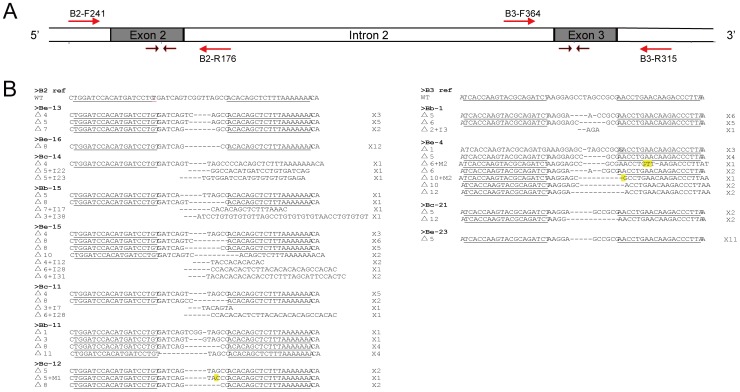
Sequences of germline mutations induced by B2 and B3. (A) Schematic representation of the structure of the *BmBlos2* gene showing part of the 1^st^ intron, 2^nd^ exon, 2^nd^ intron, 3^rd^ exon, and part of the 3^rd^ intron. The red arrows represent the primers used for PCR amplification. (B) Sequences of the mutations from different broods. The wild-type sequence is shown at the top. The marks beginning with “>” represent the brood numbers used during crossing. The “Δ” or “I” at the beginning of each line represents deletions or insertions and the following number represents the size of deletion or insertion. “M” represents substitution. The terms beginning with X at the end of each line represents the number of mutations recovered by sequence. The TALEN recognition sites are underlined, deletions are indicated by dashed lines, insertions are indicated by removal of the original sequences, and substitutions are highlighted in yellow.

To determine whether the mutation occurred at the specified locus and what types of mutations might have been induced by TALEN, we extracted the genomic DNA of 75 and 51 female mutants from 8 B2 broods and 4 B3 broods, respectively. The regions surrounding the target sites were amplified by PCR with the appropriate primer sets (Table S2) and sequenced. We identified 113 mutant sequences including small deletions, insertions, and substitutions, most of which were small deletions (Figure 3 and Figure S2). All of the mutations occurred between the TALEN binding sites. We compared the types of mutations generated by TALEN with those by ZFN [24]. We found that deletions occurred more frequently among the TALEN-induced mutations and the substitution of a few nucleotides was also more common (Figure S2). A similar phenomenon was observed when the B2 and B3 mutations were compared. These differences might have been caused by the relative activities of the different nucleases. More interestingly, the majority of deletions were longer in the B2 mutations than in the B3 mutations (Figure S2), the former of which has higher activity.

### Targeted Chromosomal Deletions using two Pairs of TALENs

**Figure 4:**
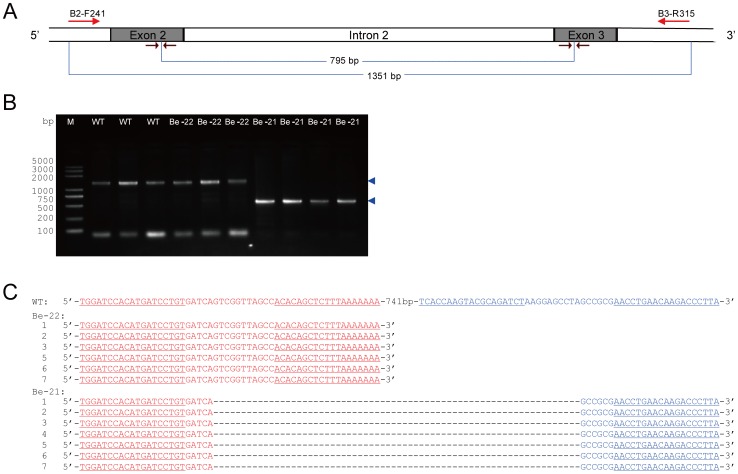
Large chromosomal deletions using two TALENs. (A) Schematic representation of the structure of partial *BmBlos2* gene as described in Figure 3. Red arrows at the top indicate primers used for PCR amplification. Blue lines at the bottom show the expected results of PCR amplification with (556 bp) or without (1351 bp) the large chromosomal deletion. (B) Gel analysis of PCR amplifications. WT represents the wild-type silkworm. Be-21 and Be-22 represent two G1 silkworm broods from G0 silkworms co-injected with B2 and B3, respectively. The numbers on the left represent the sizes of the DNA ladder (DL2000 plus). The blue arrows on the right indicate the two expected PCR products. (C) Sequences of the PCR products. The wild-type sequence is shown at the top with the full B2 site in red and the B3 site in blue. The TALEN recognition sites are underlined, and deletions are indicated by dashed lines.

Although both mosaic and germline mutations were obtained with high frequency through microinjection, deletions larger than 12 bp did not occur. In many cases of genome manipulation, the deletion of several hundred base pairs or longer are expected. To determine whether co-injection of two pairs of TALENs induces large deletions, which was demonstrated in mammalian cell lines using ZFN [25], [26], a mixture of B2 and B3 mRNAs was injected into the silkworm embryos. The experiment resulted in 20% mosaic mutations and 14% germline mutations in the G1 broods (Be-21 and Be-22, respectively). A total of 132 mutant silkworms were observed, all of which were female (Table S1). To determine whether these mutations were caused by large deletions or by a single TALEN at each locus, 7 mutant silkworms were randomly chosen from each brood. The genomic DNA of 14 mutant and 3 wild-type silkworms was extracted and the fragments containing two target sites were amplified by PCR. As expected, we observed smaller amplified DNA segments from all 7 silkworms in B2-21 (Figure 4B). The PCR products were about 550 bp in length, which was as expected if the DNA segments between two TALEN sites were deleted from the genome (Figure 4A). Sequencing of the PCR products confirmed that the left site of B2 and right site of B3 were joined and the middle segments were deleted (Figure 4C). These results indicate that TALEN can be used to produce large chromosome deletions by generating two DSBs at two distal sites in one chromosome. More importantly, these large deletions could be transmitted through the germline at a considerable frequency.

## Discussion

We reported high-frequency TALEN-induced mutagenesis in *B. mori*. Both of the constructed TALENs effectively induced heritable mosaic mutations. This finding suggests that TALENs can be used in silkworm. During the preparation of this manuscript, Liu et al. published their work in which TALENs was demonstrated to modify the *Drosophila yellow* gene [31]. Both of our work demonstrated that TALENs could work in insect species. More importantly, we have shown the induction of large chromosomal deletions using two TALENs.

More importantly, our results indicate that several issues should be carefully addressed to make a successful genome editing experiment feasible. 1) Although there are increasing publications describing strategies to assemble custom TALEs, the assembly procedures were not always easy for those who are not skilled. We showed that TALEs can be directly synthesized after optimization of codon usage. 2) In the case of manipulation of *BmBlos2*, we designed 2 pairs of TALENs targeting to the 2^nd^ and 3^rd^ exons. Although both of them can induce mutations, the frequencies are different with each other, which demonstrate that different TALENs have different cleavage activity. This indicates that to perform a successful functional knock out of a gene, one may need to design 2 TALEN targeting to the one third of the open reading frame. 3) We observed that silkworms that hatched later will generate more mosaic mutation. Although we can not completely explain this phenomenon, researchers working on insect mutagenesis can use this rule to increase the frequency and make their screening easier. 4) We demonstrate that mosaic G0 silkworms were more likely to produce mutant G1 silkworms than non-mosaic G0 silkworms. This rule can also help researchers screen mutants from a smaller population. 5) *BmBlos2* is Z chromosome located and will produce visible phenotype, so it is easy to detect mutants. For genes those are autochromosome located and don’t produce visible phenotype, other strategies such as surveyor nuclease technology or HR mediated knock in may be used.

Silkworm is an economically important insect and a model organism for studying lepidopteran and arthropod biology [32]. Genetic and physiologic studies on silkworm facilitate fundamental findings on pheromones, hormones, brain structures, physiology, and genetics of insects. As a primary producer of silk, this organism has promising potential in the production of recombinant proteins, including drugs and spider silk in place of original cocoon silk [33], [34]. The efficient genome editing presented here, together with the complete genome sequences, natural genetic resources, and established molecular tools may significantly promote the understanding and exploration of this species. Furthermore, the methods for genetic manipulation are parallel those in *Drosophila* and other insects. ZFNs have been tested in many plants, animals, and various types of mammalian cells. However, among insects, only a few studies on *Drosophila* and one in silkworm demonstrated the possibility of ZFN-induced mutagenesis [21]–[24]. The lack of experience with ZFNs and the difficulty of generating ZFNs that target a desired sequence hinder the application of genome editing in insects. Based on this study’s results, TALENs, which possess many advantages over ZFNs [35], might be applied in other insect species. The establishment of efficient genome editing methods will greatly promote insect genetic engineering, which is crucial to disease control and pest management [15].

In addition to traditional TALEN-induced targeted mutagenesis, TALENs can be used to induce more complicated mutagenesis other than micro-deletions or insertions. We showed that simultaneous expression of two pairs of TALENs generates heritable large chromosomal deletions. Targeted large chromosomal deletions are important in genome editing and have broad applications in many areas of biology, biotechnology, and gene therapy. It is indispensable in the deletion of whole gene clusters, regulatory regions or transgene markers. The generation of custom TALENs and ssODN donors enable researchers to conduct sophisticated genetic engineering with faster speed and greater freedom [27]. The aforementioned TALEN-induced chromosomal deletions may be applied to other organism or cultured cell. All the results and experiences will promote the utility and applicability of TALEN research.

## Supporting Information

Figure S1 **Frequency of TALEN induced mutations.**
(PDF)

Figure S2 **Comparison of the outcomes between TALENs and ZFNs.**
(PDF)

Table S1 **Microinjection of TALEN mRNA into the embryo of Nistari.**
(PDF)

Table S2 **Primers used in this study.**
(PDF)

Text S1 **Complete DNA and amino acid sequences of TALEN ORF.**
(PDF)

## References

[1] Robbins J (2011) Twenty Years of Gene Targeting: what we don’t know. Circ Res 109: 722–723. 21921271 10.1161/CIRCRESAHA.111.249912

[2] Caroll D (2011) Genome engineering with zinc-finger nucleases. Genetics 188: 733–782. 10.1534/genetics.111.131433 PMC3176093 21828278

[3] Urnov FD, Rebar EJ, Holmes MC, Zhang HS, Gregory PD (2010) Genome editing with engineered zinc finger nucleases. Nat Rev Genet 11: 636–646. 20717154 10.1038/nrg2842

[4] Bogdanove AJ, Voyta DJ (2011) TAL effectors: customizable proteins for DNA targeting. Science 33: 1843–1846. 10.1126/science.1204094 21960622

[5] Boch J, Scholze H, Schornack S, Landgraf A, Hahn S, et al (2009) Breaking the code of DNA binding specificity of TAL-type III effectors. Science 326: 1509–1512. 19933107 10.1126/science.1178811

[6] Christian M, Cermark T, Doyle EL, Schmidt C, Zhang F, et al (2010) TAL effector nucleases create targeted DNA double-strand breaks. Genetics 186: 757–761. 20660643 10.1534/genetics.110.120717 PMC2942870

[7] Zhang F, Cong L, Lodato S, Kosuri S, Church GM, et al (2011) Efficient construction of sequence-specific TAL effectors for modulating mammalian transcription. Nat Biotechnol 29: 149–153. 21248753 10.1038/nbt.1775 PMC3084533

[8] Li T, Huang S, Zhao X, Wright DA, Carpenter S, et al (2011) Modularly assembled designer TAL effector nucleases for targeted gene knockout and gene replacement in eukaryotes. Nucleic Acids Res 39: 6315–6325. 21459844 10.1093/nar/gkr188 PMC3152341

[9] Wood AJ, Lo TW, Zeitler B, Pickle CS, Ralston EJ, et al (2011) Targeted genome editing across species using ZFNs and TALENs. Science 333: 307. 21700836 10.1126/science.1207773 PMC3489282

[10] Tesson L, Usal C, Ménoret S, Leung E, Niles BJ, et al (2011) Knockout rats generated by embryo microinjection of TALENs. Nat Biotechnol 29: 695–696. 21822240 10.1038/nbt.1940

[11] Sander JD, Cade D, Khayter C, Reyon D, Peterson RT, et al (2011) Targeted gene disruption in somatic zebrafish cells using engineered TALENs. Nat Biotechnol 29: 697–698. 21822241 10.1038/nbt.1934 PMC3154023

[12] Huang P, Xiao A, Zhou M, Zhu Z, Lin S, et al (2011) Heritable gene targeting in zebrafish using customized TALENs. Nat Biotechnol 29: 699–700. 21822242 10.1038/nbt.1939

[13] Christian M, Cermak T, Doyle EL, Schmidt C, Zhang F, et al (2010) Targeting DNA double-strand breaks with TAL effector nucleases. Genetics 186: 757–761. 20660643 10.1534/genetics.110.120717 PMC2942870

[14] Hockemeyer D, Wang H, Kiani S, Lai CS, Gao Q, et al (2011) Genetic engineering of human pluripotent cells using TALE nucleases. Nat Biotechnol 29: 731–734. 21738127 10.1038/nbt.1927 PMC3152587

[15] Fraser MJ (2012) Insect Transgenesis: Current Applications and Future Prospects. Annu Rev Entomol 57: 267–289. 22149266 10.1146/annurev.ento.54.110807.090545

[16] Handler AM, Zimowska GJ, Horn C (2004) Post-integration stabilization of a transposon vector by terminal sequence deletion in *Drosophila* melanogaster. Nat Biotechnol 22: 1150–1154. 15300258 10.1038/nbt1002

[17] Dafa’alla TH, Condon GC, Condon KC, Phillips CE, Morrison NI, et al (2006) Transposon-free insertions for insect genetic engineering. Nat Biotechnol 24: 820–821. 16823373 10.1038/nbt1221

[18] Schetelig MF, Götschel F, Viktorinová I, Handler AM, Wimmer EA (2011) Recombination technologies for enhanced transgene stability in bioengineered insects. Genetica 139: 71–78. 20844938 10.1007/s10709-010-9494-4 PMC3030938

[19] Rong YS, Golic KG (2000) Gene Targeting by Homologous Recombination in *Drosophila* . Science 288: 2013–2017. 10856208 10.1126/science.288.5473.2013

[20] Gong WJ, Golic KG (2003) Ends-out, or replacement, gene targeting in *Drosophila* . Proc Natl Acad Sci USA 100: 2556–2561. 12589026 10.1073/pnas.0535280100 PMC151379

[21] Bibikova M, Golic M, Golic KG, Carroll D (2002) Targeted Chromosomal Cleavage and Mutagenesis in *Drosophila* Using Zinc-Finger Nucleases. Genetics 161: 1169–1175. 12136019 10.1093/genetics/161.3.1169 PMC1462166

[22] Beumer KJ, Bhattacharyya G, Bibicova M, Trautman JK, Carroll D (2006) Efficient Gene Targeting in *Drosophila* with Zinc Finger Nucleases. Genetics 172: 2391–2403. 16452139 10.1534/genetics.105.052829 PMC1456366

[23] Beumer KJ, Trautman JK, Bozas A, Liu JL, Rutter J, et al (2006) Efficient gene targeting in *Drosophila* by direct embryo injection with zinc-finger nucleases. Proc Natl Acad Sci USA 105: 19821–19826. 10.1073/pnas.0810475105 PMC2604940 19064913

[24] Takasu Y, Kobayashi I, Beumer K, Uchino K, Sezutsu H, et al (2010) Targeted mutagenesis in the silkworm *Bombyx mori* using zinc finger nuclease mRNA injection. Insect Biochem Mol Biol 40: 759–765. 20692340 10.1016/j.ibmb.2010.07.012

[25] Lee HJ, Kim E, Kim JS (2010) Targeted chromosomal deletions in human cells using zinc finger nucleases. Genome Res 20: 81–89. 19952142 10.1101/gr.099747.109 PMC2798833

[26] Lee HJ, Kweon J, Kim E, Kim S, Kim JS (2012) Targeted chromosomal duplications and inversions in the human genome using zinc finger nucleases. Genome Res 22: 539–548. 22183967 10.1101/gr.129635.111 PMC3290789

[27] Chen F, Pruett-Miller SM, Huang Y, Gjoka M, Duda K, et al (2011) High-frequency genome editing using ssDNA oligonucleotides with zinc-finger nucleases. Nat Methods 8: 753–755. 21765410 10.1038/nmeth.1653 PMC3617923

[28] Cermak C, Doyle EL, Christian M, Wang L, Zhang Y, et al (2011) Efficient Design and assembly of custom TALEN and other TAL effector-based constructs for DNA targeting. Nucleic Acids Res 39: 7879–7889. 10.1093/nar/gkr218 PMC3130291 21493687

[29] Toshiki T, Chantal T, Corinne R, Toshio K, Eappen A, et al (2000) Germline transformation of the silkworm *Bombyx mori* L. using a piggyBac transposon-derived vector. Nat Biotechnol 18: 81–84. 10625397 10.1038/71978

[30] Zhao AC, Long DP, Ma SY, Xu LX, Zhang MR, et al.. (2011) Efficient strategies for changing the diapause character of silkworm eggs and for the germline transformation of diapause silkworm strains. Insect Sci Article first published online: 28 AUG 2011 DOI: 10.1111/j.1744–7917.2011.01422.x.

[31] Liu J, Li C, Yu Z, Huang P, Wu H, et al (2012) Efficient and Specific Modifications of the Drosophila Genome by Means of an Easy TALEN Strategy. J Genet Genomics 39: 209–215. 22624882 10.1016/j.jgg.2012.04.003

[32] Goldsmith MR, Shimada T, Abe H (2005) The genetics and genomics of the silkwo*rm, Bombyx mori.* . *Annu Rev Entomol* 50 71–100. 15355234 10.1146/annurev.ento.50.071803.130456

[33] Tomita M, Munetsuna H, Sato T, Adachi T, Hino R, et al (2003) Transgenic silkworms produce recombinant human type III procollagen in cocoons. Nat Biotechnol 21: 52–56. 12483223 10.1038/nbt771

[34] Teuléa F, Miao Y, Sohn BH, Kim YS, Hull JJ, et al (2012) Silkworms transformed with chimeric silkwormspider silk genes spin composite silk fibers with improved mechanical properties. Proc Natl Acad Sci USA 109: 923–928. 22215590 10.1073/pnas.1109420109 PMC3271896

[35] DeFrancesco L (2011) Move over ZFNs. Nat Biotechnol 29: 681–684. 21822235 10.1038/nbt.1935

