# Supplementary material for: Highly Efficient and Specific Genome Editing in Silkworm Using Custom TALENs
Source: PLoS One. 2012 Sep 18;7(9):e45035. doi: 10.1371/journal.pone.0045035 (PMC3445556; doi:10.1371/journal.pone.0045035)
Supplement: Figure S1 — Frequency of TALEN induced mutations. (PDF) [file pone.0045035.s001.pdf]

Figure S1

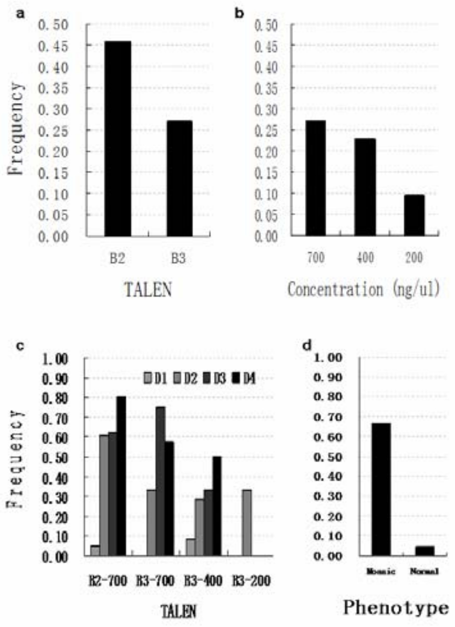

Figure S1 Frequency of TALEN induced mutations. (a) Frequency of mosaic mutations using different TALENs. B2 showed a relatively higher activity of inducing mosaic mutations than B3. (b) Frequency of B3 TALEN injected with different concentrations. The activity of B3 showed a strict dose-dependence. (c) Frequency of mosaic mutations in punctual or delayed hatched silkworms. Usually, silkworms hatch on the 10th day of embryonic development. Microinjections of plasmid or mRNA often led to a delayed hatch of some silkworms by one or two days. In all of the four injections, the delayed silkworms presented higher frequency of mosaic mutations. D1, D2, D3 and D4 represent the silkworms hatched at the 10th, 11th, 12th and 13th day, respectively. (d) Frequency of germline mutations from mosaic and wild type G0 silkworms.
