# Supplementary material for: Highly Efficient and Specific Genome Editing in Silkworm Using Custom TALENs
Source: PLoS One. 2012 Sep 18;7(9):e45035. doi: 10.1371/journal.pone.0045035 (PMC3445556; doi:10.1371/journal.pone.0045035)
Supplement: Figure S2 — Comparison of the outcomes between TALENs and ZFNs. (PDF) [file pone.0045035.s002.pdf]

Figure S2

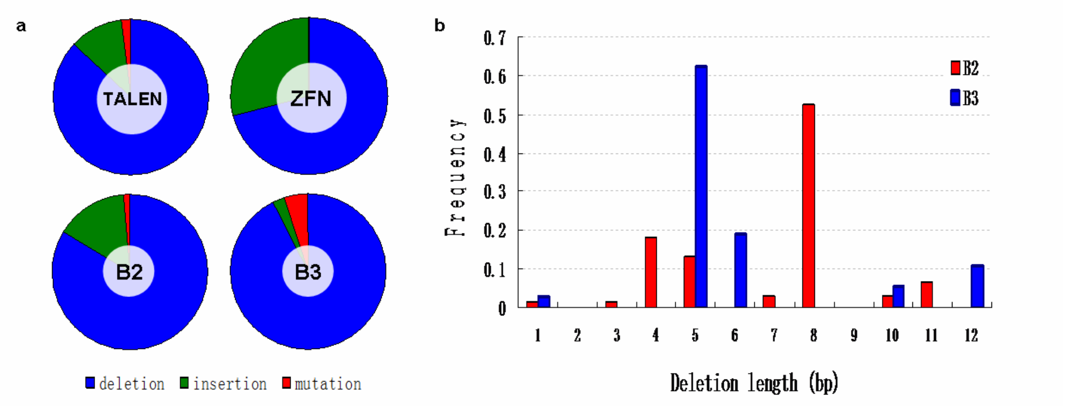

Figure S2 Comparison of the outcomes between TALENs and ZFNs. Pie charts labeled with TALEN, ZFN, B2 and B3 represent the number of mutations induced by TALEN (total of independently injected B2 or B3), ZFN, B2, and B3 respectively. The data for ZFN induced mutagenesis are from Takasu Y. et al. (2010). The bar chart represents the deletion length of B2 and B3 mutations.
