# Supplementary material for: Highly Efficient and Specific Genome Editing in Silkworm Using Custom TALENs
Source: PLoS One. 2012 Sep 18;7(9):e45035. doi: 10.1371/journal.pone.0045035 (PMC3445556; doi:10.1371/journal.pone.0045035)
Supplement: Table S1 — Microinjection of TALEN mRNA into the embryo of Nistari. (PDF) [file pone.0045035.s003.pdf]

Table S1 Microinjection of TALEN mRNA into the embryo of Nistari.

TALENs	Concn.	Injected (strain)	Hatched	5th larvae (mosaic)	Mosaic Frequency	Mosaic Pupae (♂)	G1 broods (positive)	Germline Frequency	Positive larvae	Positive pupae (♂)
B2	700 ng/ul	968 (Nistari)	225	144(66)	0.46	26(12)	29(9)	0.31	630	104(21)
B3	700 ng/ul	521 (Nistari)	101	52(14)	0.27		14(2)	0.14		
B3	400 ng/ul	331 (Nistari)	69	53(12)	0.23	9(7)	18(2)	0.11	277	85(7)
B3	200 ng/ul	299 (Nistari)	35	21(2)	0.10		9(2)	0.22		
B2+B3	350 ng/ul	326 (Nistari)	51	40(8)	0.20	5(2)	14(2)	0.14	132	4(0)

TALENs were injected into the embryos of silkworm as mRNA. The percentages shown in the sixth and ninth columns were derived using the numbers in the column to the left. The numbers in the “()” were used as numerator and the numbers to the left were used as denominator.
