# Supplementary material for: Highly Efficient and Specific Genome Editing in Silkworm Using Custom TALENs
Source: PLoS One. 2012 Sep 18;7(9):e45035. doi: 10.1371/journal.pone.0045035 (PMC3445556; doi:10.1371/journal.pone.0045035)
Supplement: Table S2 — Primers used in this study. (PDF) [file pone.0045035.s004.pdf]

Table S2 Primers used in this study.

Names	Sequences	Tm.	Purpose
>B2-F241	TTGGTCCAGTAGGTTTGAAGTAGGT	60.7	PCR amplification and sequencing
>B2-R176	ATCCTGATTAACCTAGTTTACACACAT	61.8	PCR amplification
>B3-F364	TCCAATTTGAGGGCAATGCTAC	57.7	PCR amplification and sequencing
>B3-R315	ATTTCACCACCTCATTCAACTAAGAT	60.0	PCR amplification

Sequences were written from 5’ to 3’.
