# Supplementary material for: Highly Efficient and Specific Genome Editing in Silkworm Using Custom TALENs
Source: PLoS One. 2012 Sep 18;7(9):e45035. doi: 10.1371/journal.pone.0045035 (PMC3445556; doi:10.1371/journal.pone.0045035)
Supplement: Text S1 — Complete DNA and amino acid sequences of TALEN ORF. (PDF) [file pone.0045035.s005.pdf]

Text S1 Complete DNA and amino acid sequences of TALEN ORF. The RVDs are highlighted in yellow.

>B2-L DNA sequence

ATGAGATCTGACTACAAAGACCATGACGGTGATTATAAAGATCATGACATCGATTAC

AAGGATGACGATGACAAGATGGCCCCCAAGAAGAAGAGGAAGGTGGGCATTCATGGG

GTACCCATGATGTCTCGTACTCGCTTGCCTTCGCCTCCTGCTCCTTCTCCCGCCTTC

TCCGCTGGTTCATTTTCTGACTTACTTCGCCAATTCGACCCGAGCTTATTCAACACT

AGCCTTTTTGATTCCCTGCCTCCATTCGGTGCTCACCATACAGAAGCTGCCACTGGT

GAATGGGACGAGGTTCAATCCGGACTGAGGGCAGCAGATGCACCACCACCTACCATG

AGAGTGGCTGTTACGGCTGCCAGGCCACCGAGAGCTAAACCAGCACCAAGAAGGCGT

GCAGCACAGCCATCAGACGCTTCTCCGGCTGCCCAAGTGGATCTCAGGACCTTAGGT

TACTCACAACAGCAACAGGAAAAAATCAAGCCAAAAGTTAGATCTACAGTCGCCCAG

CACCATGAGGCATTGGTGGGTCACGGATTTACTCACGCGCATATTGTTGCTCTGTCG

CAGCATCCGGCAGCGTTGGGAACCGTCGCTGTAAAGTATCAAGACATGATAGCTGCC

CTCCCCGAAGCCACACACGAGGCAATCGTGGGCGTTGGTAAACAATGGTCAGGCGCT

AGGGCCTTGGAAGCGCTGTTGACAGTGGCTGGAGAGCTCAGAGGCCCCCCTTTACAA

CTTGATACCGGTCAGCTCTTAAAGATAGCTAAACGCGGTGGAGTCACGGCAGTAGAA

GCGGTGCACGCTTGGCGTAACGCGTTAACAGGAGCTCCCCTGAATTTGACTCCTGAA

CAGGTGGTTGCAATCGCGTCGAACAATGGCGGTAAACAAGCCCTTGAGACCGTACAG

CGCCTTCTGCCAGTGTTGTGCCAAGCACACGGACTCACGCCGCAACAGGTCGTAGCT

ATTGCCAGTAACAATGGAGGCAAACAAGCGTTAGAAACAGTTCAGCGTTTGCTCCCC

GTCTTATGTCAAGCTCATGGCCTTACTCCTCAGCAAGTGGTTGCAATCGCGTCAAAC

ATTGGTGGAAAGCAAGCCTTGGAGACCGTACAGAGGTTACTTCCAGTGCTGTGCCAA

GCACACGGTTTGACGCCGGAACAGGTCGTAGCTATAGCCTCTAATGGCGGTGGAAAA

CAAGCGCTCGAGACAGTTCAGAGACTGTTGCCCGTCCTCTGTCAAGCACACGGATTA

ACACCAGAGCAAGTTGTCGCAATCGCGAGCCATGACGGCGGTAAACAAGCCCTGGAG
ACCGTACAGGCACTCTTACCAGTGCTTTGCCAAGCACACGGACTGACGCCAGAGCAA
GTCGTAGCTATTGCCTCCCATGACGGTGGCAAACAAGCGTTGGAGACAGTTCAGGCT
CTTCTGCCCGTCTTGTGTCAAGCTCACGGTCTCACACCAGAACAGGTGGTTGCAATA
GCGTCGAACATCGGTGGAAAGCAAGCCCTCGAGACCGTACAGGCATTGCTCCCAGTG
TTATGCCAAGCTCACGGACTTACTCCTGAGCAGGTTGTAGCTATTGCCAGTCACGAT
GGCGGTAAACAAGCTCTGGAGACAGTTCAGCGCTTACTTCCCGTCCTGTGTCAAGCC
CATGGCCTGACACCCGAGCAGGTGGTTGCAATTGCGTCAAACATAGGAGGCAAGCAA
GCATTGGAGACCGTACAGCGTCTGTTGCCAGTGCTCTGCCAGGCTCACGGTTTAACT
CCTCAGCAAGTTGTTGCTATAGCCTCTAACGGTGGAGGCAAACAAGCCCTCGAAACA
GTTCAGAGGCTCTTACCCGTCCTTTGTCAAGCTCATGGACTTACACCCGAACAAGTT
GTGGCAATCGCGAGCAACAATGGTGGAAAGCAAGCGCTGGAGACCGTACAGGCTCTT
CTGCCAGTCTTGTGCCAAGCTCACGGCCTCACTCCTGAACAGGTCGTAGCTATCGCC
TCCAACATTGGCGGTAAACAAGCCTTGGAGACAGTGCAGAGATTGCTCCCCGTTTTA
TGTCAAGCTCATGGTCTTACACCCGAACAAGTCGTTGCAATTGCGTCGAATGGAGGT
GGTAAACAGGCCCTGGAGACCGTCCAGCGCTTGTTGCCAGTGCTGTGCCAAGCTCAC
GGACTGACTCCAGAGCAGGTTGTTGCTATAGCCAGTCATGACGGAGGCAAACAGGCT
TTGGAGACAGTTCAGCGTTTGCTGCCTGTTCTCTGTCAAGCTCACGGCTTAACACCC
GAACAAGTTGTTGCAATCGCGTCACATGATGGTGGAAAGCAAGCCCTGGAGACCGTG
CAGAGACTCTTACCAGTTCTTTGCCAAGCTCACGGTCTGACTCCTCAGCAAGTTGTC
GCTATTGCATCTAATGGAGGTGGAAGGCCAGCACTGGAAACAGTCCAACGTCTTCTG
CCTGTGTTGTGTCAGGCTCATGGACTCACTCCAGAACAAGTGGTTGCAATAGCGAGC
AATAACGGTGGTAAACAGGCTTTGGAGACCGTTCAGCGCTTGCTCCCGGTCTTATGC
CAGGCTCACGGCCTTACGCCCCAGCAAGTTGTTGCTATTGCATCCAACGGAGGTGGT
AGGCCTGCACTCGAATCGATAGTGGCACAATTAAGTCGTCCCGACCCTGCCCTTGCA
GCGCTGACTAATGATCACTTGGTCGCACTCGCGTGCTTAGGAGGCAGACCTGCCCTT

GATGCAGTGAAAAAGGGTCTGCCACATGCTCCCGCTCTCATCAAAAGAACCAATCGC

CGTATTCCCGAACGCACCTCGCACCGTGTGGCTGGATCCCAGCTGGTGAAGAGCGAG

CTGGAGGAGAAGAAGTCCGAGCTGCGGCACAAGCTGAAGTACGTGCCCCACGAGTAC

ATCGAGCTGATCGAGATCGCCAGGAACAGCACCCAGGACCGCATCCTGGAGATGAAG

GTGATGGAGTTCTTCATGAAGGTGTACGGCTACAGGGGAAAGCACCTGGGCGGAAGC

AGAAAGCCTGACGGCGCCATCTATACAGTGGGCAGCCCCATCGATTACGGCGTGATC

GTGGACACAAAGGCCTACAGCGGCGGCTACAATCTGCCTATCGGCCAGGCCGACGAG

ATGCAGAGATACGTGAAGGAGAACCAGACCCGGAATAAGCACATCAACCCCAACGAG

TGGTGGAAGGTGTACCCTAGCAGCGTGACCGAGTTCAAGTTCCTGTTCGTGAGCGGC

CACTTCAAGGGCAACTACAAGGCCCAGCTGACCAGGCTGAACCACAAAACCAACTGC

AATGGCGCCGTGCTGAGCGTGGAGGAGCTGCTGATCGGCGGCGAGATGATCAAAGCC

GGCACCCTGACACTGGAGGAGGTGCGGCGCAAGTTCAACAACGGCGAGATCAACTTC

TGATAA

>B2-L amino acid sequence

MRSDYKDHDGDYKDHDIDYKDDDDKMAPKKKRKVGIHGVPMMSRTRLPSPPAPSPAF

SAGSFSDLLRQFDPSLFNTSLFDSLPPFGAHHTEAATGEWDEVQSGLRAADAPPPTM

RVAVTAARPPRAKPAPRRRAAQPSDASPAAQVDLRTLGYSQQQQEKIKPKVRSTVAQ

HHEALVGHGFTHAHIVALSQHPAALGTVAVKYQDMIAALPEATHEAIVGVGKQWSGA

RALEALLTVAGELRGPPLQLDTGQLLKIAKRGGVTAVEAVHAWRNALTGAPLNLTPE

QVVAIASNNGGKQALETVQRLLPVLCQAHGLTPQQVVAIASNNGGKQALETVQRLLP

VLCQAHGLTPQQVVAIASNIGGKQALETVQRLLPVLCQAHGLTPEQVVAIASNGGGK

QALETVQRLLPVLCQAHGLTPEQVVAIASHDGGKQALETVQALLPVLCQAHGLTPEQ

VVAIASHDGGKQALETVQALLPVLCQAHGLTPEQVVAIASNIGGKQALETVQALLPV

LCQAHGLTPEQVVAIASHDGGKQALETVQRLLPVLCQAHGLTPEQVVAIASNIGGKQ

ALETVQRLLPVLCQAHGLTPQQVVAIASNGGGKQALETVQRLLPVLCQAHGLTPEQV

VAIASNNGGKQALETVQALLPVLCQAHGLTPEQVVAIASNIGGKQALETVQRLLPVL

CQAHGLTPEQVVAIASNGGGKQALETVQRLLPVLCQAHGLTPEQVVAIASHDGGKQA

LETVQRLLPVLCQAHGLTPEQVVAIASHDGGKQALETVQRLLPVLCQAHGLTPQQVV

AIASNGGGRPALETVQRLLPVLCQAHGLTPEQVVAIASNNGGKQALETVQRLLPVLC

QAHGLTPQQVVAIASNGGGRPALESIVAQLSRPDPALAALTNDHLVALACLGGRPAL

DAVKKGLPHAPALIKRTNRRIPERTSHRVAGSQLVKSELEEKKSELRHKLKYVPHEY

IELIEIARNSTQDRILEMKVMEFFMKVYGYRGKHLGGSRKPDGAIYTVGSPIDYGVI

VDTKAYSGGYNLPIGQADEMQRYVKENQTRNKHINPNEWWKVYPSSVTEFKFLFVSG

HFKGNYKAQLTRLNHKTNCNGAVLSVEELLIGGEMIKAGTLTLEEVRRKFNNGEINF

>B2-R DNA sequence

ATGGACTACAAAGACCATGACGGTGATTATAAAGATCATGACATCGATTACAAGGAT

GACGATGACAAGATGGCCCCCAAGAAGAAGAGGAAGGTGGGCATCCACGGGGTACCC

ATGATGTCGCGCACAAGATTGCCAAGCCCACCAGCACCTTCACCAGCATTCTCAGCG

GGTTCCTTTTCCGATTTACTCCGTCAGTTCGACCCGTCGTTATTCAACACTTCGCTT

TTTGATAGTCTGCCTCCATTCGGTGCTCACCATACAGAAGCTGCCACTGGAGAATGG

GACGAGGTTCAAAGTGGACTGAGGGCAGCAGATGCACCACCACCTACCATGAGAGTG

GCTGTTACGGCTGCAAGGCCACCAAGAGCTAAGCCAGCACCAAGAAGGCGTGCAGCA

CAGCCAAGCGACGCTTCCCCAGCTGCACAAGTGGATCTCAGGACCTTAGGTTACAGC

CAACAGCAACAGGAAAAAATCAAGCCAAAAGTTAGATCCACAGTCGCCCAGCACCAT

GAGGCATTGGTGGGACACGGCTTTACTCACGCGCATATTGTTGCTCTGTCACAGCAT

CCGGCAGCGTTGGGAACCGTCGCTGTAAAGTATCAAGACATGATAGCTGCCCTCCCC

GAAGCCACACACGAGGCAATCGTGGGTGTTGGAAAACAATGGTCAGGTGCTAGGGCC

TTGGAAGCGCTGTTGACAGTGGCTGGCGAGCTCAGAGGTCCCCCTTTACAACTTGAT

ACCGGACAGCTCTTAAAGATAGCTAAACGCGGTGGAGTCACGGCAGTAGAAGCGGTG

CACGCTTGGCGTAACGCGTTAACAGGAGCTCCCCTGAATTTGACTCCTGAACAGGTG

GTTGCAATCGCGTCAAACGGCGGTGGAAAGCAAGCCCTTGAGACCGTACAGCGCCTT
CTGCCAGTGTTGTGCCAAGCACACGGCCTCACGCCGCAACAGGTCGTAGCTATTGCC
TCTAATGGCGGTGGAAAACAAGCGTTAGAAACAGTTCAGCGTTTGCTCCCCGTCTTA
TGTCAAGCTCATGGTCTTACTCCTCAGCAAGTGGTTGCAATCGCGAGCATTGGTGGA
GGAAAGCAAGCCTTGGAGACCGTACAGAGGTTACTTCCAGTGCTGTGCCAAGCACAC
GGATTGACGCCGGAACAGGTCGTAGCTATAGCCTCCAACGGTGGAGGAAAACAAGCG
CTCGAGACAGTTCAGAGACTGTTGCCCGTCCTCTGTCAAGCACATGGCTTAACACCA
GAGCAAGTTGTCGCAATCGCGTCGAATGGTGGCGGAAAGCAAGCCCTGGAGACCGTA
CAGGCACTCTTACCAGTGCTTTGCCAAGCCCACGGTCTTACTCCTGAGCAAGTTGTA
GCTATTGCCAGTAACGGTGGCGGAAAACAAGCGTTGGAGACAGTTCAGGCTCTTCTG
CCCGTCTTGTGTCAAGCTCATGGACTCACACCAGAACAGGTGGTTGCAATAGCGTCA
AATATCGGCGGTAAACAAGCCCTCGAGACCGTACAGGCATTGCTCCCAGTGTTATGC
CAAGCTCACGGCCTTACTCCTGAGCAAGTGGTTGCTATTGCCTCTAACATAGGAGGC
AAACAAGCTCTGGAGACAGTTCAGCGCTTACTTCCCGTCCTGTGTCAAGCCCATGGA
CTGACACCAGAGCAAGTTGTGGCAATCGCGAGCAATATTGGTGGAAAGCAAGCATTG
GAGACCGTACAGCGTCTGTTGCCAGTGCTCTGCCAGGCTCACGGATTAACTCCTCAG
CAAGTTGTTGCTATAGCCTCCAACAATGGTGGTAAACAGGCCCTCGAAACAGTTCAG
AGGCTCTTACCCGTCCTTTGTCAAGCTCATGGCCTTACACCCGAGCAGGTTGTCGCA
ATCGCGTCAAACTCTGGAGGCAAGCAAGCGCTGGAGACCGTACAGGCTCTTCTGCCA
GTCTTGTGCCAAGCTCACGGTCTCACTCCTGAGCAAGTCGTAGCTATTGCCTCGAAT
AAGGGTGGAAAACAAGCCTTGGAGACAGTGCAGAGATTGCTCCCCGTTTTATGTCAA
GCTCACGGACTTACACCCGAGCAGGTCGTTGCAATAGCGAGTCATGATGGTGGTAAA
CAAGCCCTGGAGACCGTCCAGCGCTTGTTGCCAGTGCTGTGCCAAGCTCATGGCCTG
ACTCCTGAGCAGGTCGTAGCTATCGCCTCAAACGGAGGCGGTAAACAGGCTTTGGAG
ACAGTTCAGCGTTTGCTGCCTGTTCTCTGTCAAGCTCACGGTTTAACACCCGAACAG
GTGGTTGCAATTGCGTCTAACAATGGAGGCAAACAGGCTCTGGAGACCGTGCAGAGA

CTCTTACCAGTTCTTTGCCAAGCTCATGGACTGACTCCTCAGCAAGTCGTAGCTATA

GCAAGCAATGGTGGAGGAAGGCCAGCACTGGAAACAGTCCAACGTCTTCTGCCTGTG

TTGTGTCAGGCTCACGGCCTCACTCCAGAACAAGTGGTTGCAATTGCGTCCAACAAT

GGTGGAAAACAGGCACTGGAGACCGTTCAGCGCTTGCTCCCGGTCTTATGCCAGGCT

CACGGACTTACGCCCCAGCAAGTGGTCGCTATTGCATCGAACGGAGGTGGAAGGCCT

GCACTCGAATCAATAGTGGCACAATTATCTCGTCCCGACCCTGCCCTTGCAGCGCTG

ACTAATGATCACTTGGTCGCACTCGCGTGCTTAGGCGGTAGACCTGCCCTTGATGCA

GTGAAAAAGGGTCTGCCACATGCTCCCGCACTGATAAAACGCACCAACCGTCGTATT

CCTGAACGCACCTCACATCGTGTCGCAGGATCCCAGCTGGTGAAGAGCGAGCTGGAG

GAGAAGAAGTCCGAGCTGCGGCACAAGCTGAAGTACGTGCCCCACGAGTACATCGAG

CTGATCGAGATCGCCAGGAACAGCACCCAGGACCGCATCCTGGAGATGAAGGTGATG

GAGTTCTTCATGAAGGTGTACGGCTACAGGGGAAAGCACCTGGGCGGAAGCAGAAAG

CCTGACGGCGCCATCTATACAGTGGGCAGCCCCATCGATTACGGCGTGATCGTGGAC

ACAAAGGCCTACAGCGGCGGCTACAATCTGCCTATCGGCCAGGCCGACGAGATGGAG

AGATACGTGGAGGAGAACCAGACCCGGAATAAGCACCTCAACCCCAACGAGTGGTGG

AAGGTGTACCCTAGCAGCGTGACCGAGTTCAAGTTCCTGTTCGTGAGCGGCCACTTC

AAGGGCAACTACAAGGCCCAGCTGACCAGGCTGAACCACATCACCAACTGCAATGGC

GCCGTGCTGAGCGTGGAGGAGCTGCTGATCGGCGGCGAGATGATCAAAGCCGGCACC

CTGACACTGGAGGAGGTGCGGCGCAAGTTCAACAACGGCGAGATCAACTTC

>B2-R amino acid sequence

MDYKDHDGDYKDHDIDYKDDDDKMAPKKKRKVGIHGVPMMSRTRLPSPPAPSPAFSA

GSFSDLLRQFDPSLFNTSLFDSLPPFGAHHTEAATGEWDEVQSGLRAADAPPPTMRV

AVTAARPPRAKPAPRRRAAQPSDASPAAQVDLRTLGYSQQQQEKIKPKVRSTVAQHH

EALVGHGFTHAHIVALSQHPAALGTVAVKYQDMIAALPEATHEAIVGVGKQWSGARA

LEALLTVAGELRGPPLQLDTGQLLKIAKRGGVTAVEAVHAWRNALTGAPLNLTPEQV

VAIASNGGGKQALETVQRLLPVLCQAHGLTPQQVVAIASNGGGKQALETVQRLLPVL

CQAHGLTPQQVVAIASIGGGKQALETVQRLLPVLCQAHGLTPEQVVAIASNGGGKQA

LETVQRLLPVLCQAHGLTPEQVVAIASNGGGKQALETVQALLPVLCQAHGLTPEQVV

AIASNGGGKQALETVQALLPVLCQAHGLTPEQVVAIASNIGGKQALETVQALLPVLC

QAHGLTPEQVVAIASNIGGKQALETVQRLLPVLCQAHGLTPEQVVAIASNIGGKQAL

ETVQRLLPVLCQAHGLTPQQVVAIASNNGGKQALETVQRLLPVLCQAHGLTPEQVVA

IASNSGGKQALETVQALLPVLCQAHGLTPEQVVAIASNKGGKQALETVQRLLPVLCQ

AHGLTPEQVVAIASHDGGKQALETVQRLLPVLCQAHGLTPEQVVAIASNGGGKQALE

TVQRLLPVLCQAHGLTPEQVVAIASNNGGKQALETVQRLLPVLCQAHGLTPQQVVAI

ASNGGGRPALETVQRLLPVLCQAHGLTPEQVVAIASNNGGKQALETVQRLLPVLCQA

HGLTPQQVVAIASNGGGRPALESIVAQLSRPDPALAALTNDHLVALACLGGRPALDA

VKKGLPHAPALIKRTNRRIPERTSHRVAGSQLVKSELEEKKSELRHKLKYVPHEYIE

LIEIARNSTQDRILEMKVMEFFMKVYGYRGKHLGGSRKPDGAIYTVGSPIDYGVIVD

TKAYSGGYNLPIGQADEMERYVEENQTRNKHLNPNEWWKVYPSSVTEFKFLFVSGHF

KGNYKAQLTRLNHITNCNGAVLSVEELLIGGEMIKAGTLTLEEVRRKFNNGEINF

>B3-L DNA sequence

ATGAGATCTGACTACAAAGACCATGACGGTGATTATAAAGATCATGACATCGATTAC

AAGGATGACGATGACAAGATGGCCCCCAAGAAGAAGAGGAAGGTGGGCATTCATGGG

GTACCCATGATGAGCAGAACCAGACTCCCCAGCCCACCAGCGCCCTCGCCAGCGTTT

TCGGCAGGCAGCTTTTCGGACTTGTTACGTCAGTTTGACCCAAGCTTATTCAACACT

AGCCTTTTTGATTCCCTGCCTCCATTCGGTGCTCACCATACAGAAGCTGCCACTGGA

GAATGGGACGAGGTTCAATCCGGACTGAGGGCAGCAGATGCACCACCACCTACCATG

CGTGTGGCAGTTACGGCTGCCAGGCCACCGAGAGCTAAACCAGCACCAAGAAGGCGT

GCAGCACAACCATCAGACGCTTCTCCGGCTGCCCAGGTGGATCTCCGCACCTTAGGT

TACTCACAACAGCAACAGGAAAAAATTAAGCCGAAAGTTCGTTCTACAGTCGCCCAA

CACCATGAGGCATTGGTGGGACACGGCTTTACTCACGCGCATATAGTTGCTCTGTCG

CAACATCCGGCAGCGTTGGGAACCGTCGCTGTAAAGTATCAGGACATGATCGCTGCC

CTCCCTGAAGCCACACACGAGGCAATTGTGGGTGTTGGAAAACAGTGGTCAGGTGCT

CGCGCCTTGGAAGCGCTGTTGACAGTGGCTGGAGAGCTCCGTGGTCCACCTTTACAA

CTTGATACCGGACAGCTCTTAAAGATCGCTAAAAGGGGTGGAGTCACGGCAGTAGAA

GCGGTGCACGCTTGGAGAAACGCGTTAACAGGAGCTCCCCTGAATTTGACTCCTGAA

CAAGTGGTTGCAATTGCGTCGCACGACGGCGGTAAACAAGCTCTTGAGACCGTACAG

AGGCTTCTGCCAGTGTTGTGCCAAGCACATGGACTCACGCCAGCACAGGTCGTAGCT

ATCGCCAGTAATATTGGAGGCAAACAAGCGTTAGAAACAGTTCAGAGATTGCTCCCC

GTCTTATGTCAAGCTCACGGTCTTACTCCTGACCAGGTGGTTGCAATAGCGTCACAT

GATGGTGGAAAGCAAGCTTTGGAGACCGTACAGCGCTTACTTCCAGTGCTGTGCCAA

GCGCACGGATTGACGCCGGCTCAAGTTGTAGCTATCGCCTCTCATGATGGCGGTAAA

CAAGCCCTCGAAACAGTTCAGCGTCTGTTGCCCGTCCTCTGTCAAGCACACGGCTTA

ACTCCTGAACAGGTTGTCGCAATAGCGAGCAACATCGGAGGCAAGCAAGCGCTGGAG

ACCGTACAGAGGCTCTTACCAGTGCTTTGCCAAGCTCATGGTCTGACGCCGGACCAG

GTCGTAGCTATTGCCTCCAATATAGGTGGAAAACAAGCCTTGGAGACAGTTCAGAGA

CTTCTGCCCGTCTTGTGTCAAGCACACGGACTCACTCCTGATCAGGTTGTCGCAATT

GCGTCGAACAATGGCGGTAAACAAGCGCTCGAAACCGTACAGCGCTTGCTCCCAGTG

TTATGCCAAGCACATGGCCTTACGCCGGAACAGGTCGTAGCTATAGCCAGTAACGGA

GGCGGTAAACAAGCCCTGGAGACAGTTCAGCGTTTACTTCCCGTCCTGTGTCAAGCT

CACGGTTTGACACCAGACCAGGTGGTTGCAATCGCGTCAAATATTGGAGGCAAGCAA

GCCTTAGAAACCGTACAGAGGCTGTTGCCAGTGCTCTGCCAAGCTCACGGATTAACT

CCTGAGCAAGTCGTTGCAATCGCCTCTCATGATGGTGGAAAACAAGCCCTCGAGACA

GTTCAGAGACTCTTACCCGTCCTTTGTCAAGCTCACGGCCTGACTCCTCAACAGGTG

GTTGCAATTGCGAGCAATAACGGTGGTAAACAGGCCCTGGAAACCGTACAGCGCCTT

CTGCCAGTCTTGTGCCAAGCTCACGGTCTCACTCCTGAGCAAGTCGTAGCTATAGCC

TCCCATGACGGAGGCAAACAGGCTTTGGAGACAGTGCAGCGTTTGCTCCCCGTTTTA

TGTCAAGCTCATGGACTTACTCCTGATCAAGTTGTTGCAATAGCGTCGAACATCGGT

GGAAAGCAAGCTCTGGAGACCGTCCAGAGGTTGTTGCCAGTGCTGTGCCAAGCACAC

GGATTGACTCCTGCACAAGTTGTAGCTATTGCCAGTAATAACGGCGGTAAACAAGCC

TTGGAAACAGTTCAGCGCTTGCTGCCTGTTCTCTGTCAAGCTCATGGTTTAACTCCT

GAGCAAGTTGTCGCAATTGCGTCAAACATAGGTGGCAAGCAGGCCCTGGAGACCGTG

CAGCGTCTCTTACCAGTTCTTTGCCAAGCTCACGGACTGACGCCGCAACAAGTGGTA

GCTATTGCCTCTAATGGTGGAGGCAAACAAGCCCTGGAAACAGTCCAGAGACTTCTG

CCCGTGTTGTGTCAAGCTCACGGCCTCACTCCTGAACAGGTTGTGGCAATCGCGAGC

CATGACGGTGGAAAGCAGGCTTTGGAGACCGTTCAGCGCTTGCTCCCAGTCTTATGC

CAAGCTCACGGATTGACTCCTGAGCAGGTCGTAGCTATAGCATCCAACGGAGGTGGA

AGGCCAGCACTCGAGTCGATCGTGGCTCAATTAAGTAGACCCGACCCTGCCCTTGCA

GCGCTGACTAATGATCACTTGGTCGCACTCGCGTGCTTAGGCGGTAGACCCGCCCTT

GATGCAGTGAAAAAGGGTCTGCCACATGCTCCAGCACTCATCAAAAGAACCAATCGT

CGTATTCCCGAGAGGACATCACACAGGGTGGCGGGATCCCAGCTGGTGAAGAGCGAG

CTGGAGGAGAAGAAGTCCGAGCTGCGGCACAAGCTGAAGTACGTGCCCCACGAGTAC

ATCGAGCTGATCGAGATCGCCAGGAACAGCACCCAGGACCGCATCCTGGAGATGAAG

GTGATGGAGTTCTTCATGAAGGTGTACGGCTACAGGGGAAAGCACCTGGGCGGAAGC

AGAAAGCCTGACGGCGCCATCTATACAGTGGGCAGCCCCATCGATTACGGCGTGATC

GTGGACACAAAGGCCTACAGCGGCGGCTACAATCTGCCTATCGGCCAGGCCGACGAG

ATGCAGAGATACGTGAAGGAGAACCAGACCCGGAATAAGCACATCAACCCCAACGAG

TGGTGGAAGGTGTACCCTAGCAGCGTGACCGAGTTCAAGTTCCTGTTCGTGAGCGGC

CACTTCAAGGGCAACTACAAGGCCCAGCTGACCAGGCTGAACCACAAAACCAACTGC

AATGGCGCCGTGCTGAGCGTGGAGGAGCTGCTGATCGGCGGCGAGATGATCAAAGCC

GGCACCCTGACACTGGAGGAGGTGCGGCGCAAGTTCAACAACGGCGAGATCAACTTC

TGATAA

>B3-L amino acid sequence

MRSDYKDHDGDYKDHDIDYKDDDDKMAPKKKRKVGIHGVPMMSRTRLPSPPAPSPAF

SAGSFSDLLRQFDPSLFNTSLFDSLPPFGAHHTEAATGEWDEVQSGLRAADAPPPTM

RVAVTAARPPRAKPAPRRRAAQPSDASPAAQVDLRTLGYSQQQQEKIKPKVRSTVAQ

HHEALVGHGFTHAHIVALSQHPAALGTVAVKYQDMIAALPEATHEAIVGVGKQWSGA

RALEALLTVAGELRGPPLQLDTGQLLKIAKRGGVTAVEAVHAWRNALTGAPLNLTPE

QVVAIASHDGGKQALETVQRLLPVLCQAHGLTPAQVVAIASNIGGKQALETVQRLLP

VLCQAHGLTPDQVVAIASHDGGKQALETVQRLLPVLCQAHGLTPAQVVAIASHDGGK

QALETVQRLLPVLCQAHGLTPEQVVAIASNIGGKQALETVQRLLPVLCQAHGLTPDQ

VVAIASNIGGKQALETVQRLLPVLCQAHGLTPDQVVAIASNNGGKQALETVQRLLPV

LCQAHGLTPEQVVAIASNGGGKQALETVQRLLPVLCQAHGLTPDQVVAIASNIGGKQ

ALETVQRLLPVLCQAHGLTPEQVVAIASHDGGKQALETVQRLLPVLCQAHGLTPQQV

VAIASNNGGKQALETVQRLLPVLCQAHGLTPEQVVAIASHDGGKQALETVQRLLPVL

CQAHGLTPDQVVAIASNIGGKQALETVQRLLPVLCQAHGLTPAQVVAIASNNGGKQA

LETVQRLLPVLCQAHGLTPEQVVAIASNIGGKQALETVQRLLPVLCQAHGLTPQQVV

AIASNGGGKQALETVQRLLPVLCQAHGLTPEQVVAIASHDGGKQALETVQRLLPVLC

QAHGLTPEQVVAIASNGGGRPALESIVAQLSRPDPALAALTNDHLVALACLGGRPAL

DAVKKGLPHAPALIKRTNRRIPERTSHRVAGSQLVKSELEEKKSELRHKLKYVPHEY

IELIEIARNSTQDRILEMKVMEFFMKVYGYRGKHLGGSRKPDGAIYTVGSPIDYGVI

VDTKAYSGGYNLPIGQADEMQRYVKENQTRNKHINPNEWWKVYPSSVTEFKFLFVSG

HFKGNYKAQLTRLNHKTNCNGAVLSVEELLIGGEMIKAGTLTLEEVRRKFNNGEINF

>B3-R DNA sequence

ATGGACTACAAAGACCATGACGGTGATTATAAAGATCATGACATCGATTACAAGGAT

GACGATGACAAGATGGCCCCCAAGAAGAAGAGGAAGGTGGGCATCCACGGGGTACCC

ATGATGTCTCGCACTCGTCTCCCTAGCCCTCCAGCACCGTCACCAGCATTCTCGGCA

GGTTCCTTCTCGGACTTATTACGCCAGTTTGACCCGAGCTTATTCAATACTAGCCTT

TTTGATTCCCTGCCTCCATTCGGTGCTCACCATACAGAAGCTGCCACTGGCGAATGG

GACGAGGTTCAATCCGGTCTGAGGGCAGCAGATGCACCACCACCTACCATGAGAGTG

GCTGTTACGGCTGCCAGGCCACCGAGAGCTAAACCAGCACCAAGAAGGCGTGCAGCA

CAGCCATCAGACGCTTCTCCGGCTGCCCAAGTGGATCTCAGGACCTTAGGTTACTCA

CAACAGCAACAGGAAAAAATCAAACCAAAAGTTAGATCTACAGTCGCCCAGCACCAT

GAGGCATTGGTGGGCCACGGTTTTACTCACGCGCATATCGTTGCTCTGTCGCAGCAT

CCGGCAGCGTTGGGAACCGTCGCTGTAAAGTATCAAGACATGATTGCTGCCCTCCCC

GAAGCCACACACGAGGCAATAGTGGGAGTTGGCAAACAATGGTCAGGAGCTAGGGCC

TTGGAAGCGCTGTTGACAGTGGCTGGAGAGCTCAGAGGACCCCCTTTACAACTTGAT

ACCGGCCAGCTCTTAAAGATTGCTAAACGCGGTGGAGTCACGGCAGTAGAAGCGGTG

CACGCTTGGCGTAACGCGTTAACAGGAGCTCCCCTGAATTTGACTCCTGAACAGGTG

GTTGCAATAGCGTCGCACGACGGCGGTAAACAAGCCCTTGAGACCGTACAGCGCCTT

CTGCCAGTGTTGTGCCAAGCACATGGTCTCACGCCGCAACAGGTCGTAGCTATCGCC

AGTAACATTGGAGGCAAACAAGCGTTAGAAACAGTTCAGCGTTTGCTCCCCGTCTTA

TGTCAAGCTCACGGACTTACTCCTCAACAGGTGGTTGCAATCGCGTCAAACAATGGT

GGAAAGCAAGCCTTGGAGACCGTACAGAGGTTACTTCCAGTGCTGTGCCAAGCACAT

GGCTTGACGCCGGAACAGGTCGTAGCTATTGCCTCTAATAAGGGCGGTAAACAAGCG

CTCGAGACAGTTCAGAGACTGTTGCCCGTCCTCTGTCAAGCACACGGTTTAACACCA

GAGCAAGTGGTTGCAATAGCGAGCAACAATGGAGGCAAACAAGCCCTGGAGACCGTA

CAGGCACTCTTACCAGTGCTTTGCCAAGCCCATGGACTTACTCCTGAGCAAGTTGTA

GCTATCGCCTCCAACGGTGGAGGCAAGCAAGCGTTGGAGACAGTTCAGGCTCTTCTG

CCCGTCTTGTGTCAAGCTCACGGCCTCACACCAGAACAGGTGGTTGCAATTGCGTCG

CATGATGGTGGAAAACAAGCCCTCGAGACCGTACAGGCATTGCTCCCAGTGTTATGC

CAAGCTCACGGTCTTACTCCTGAACAGGTCGTAGCTATAGCCAGTAACGGCGGTGGA

AAGCAAGCTCTGGAGACAGTTCAGCGCTTACTTCCCGTCCTGTGTCAAGCCCATGGA

CTGACACCCGAGCAAGTTGTTGCAATCGCGTCAAATGGCGGTGGAAAACAAGCATTG

GAGACCGTACAGCGTCTGTTGCCAGTGCTCTGCCAGGCTCACGGCTTAACTCCTCAG

CAAGTTGTTGCTATTGCCTCTAACAATGGCGGTAAACAAGCCCTCGAAACAGTTCAG

AGGCTCTTACCCGTCCTTTGTCAAGCTCATGGTCTCACACCCGAGCAGGTTGTCGCA

ATAGCGAGCAACGGAGGCGGTAAACAAGCGCTGGAGACCGTACAGGCTCTTCTGCCA

GTCTTGTGCCAAGCTCACGGACTCACTCCAGAGCAAGTCGTAGCTATCGCCTCCAAT

GGAGGCGGTAAACAAGCCTTGGAGACAGTGCAGAGATTGCTCCCCGTTTTATGTCAA

GCTCACGGCCTTACACCCGAGCAGGTTGTGGCTATTGCGTCGCATGACGGTGGCAAA

CAGGCCCTGGAGACCGTCCAGCGCTTGTTGCCAGTGCTGTGCCAAGCTCATGGTCTG

ACTCCAGAGCAGGTTGTTGCTATAGCCAGTAACATCGGAGGAAAGCAGGCCTTGGAG

ACAGTTCAGCGTTTGCTGCCTGTTCTCTGTCAAGCTCACGGATTAACACCCGAGCAG

GTTGTTGCAATAGCGTCAAATAACGGTGGTAAACAGGCTTTGGAGACCGTGCAGAGA

CTCTTACCAGTTCTTTGCCAAGCTCATGGCCTGACTCCTCAGCAAGTGGTTGCTATC

GCCTCTAACAAAGGAGGCCGCCCCGCCCTGGAAACAGTCCAACGTCTTCTGCCTGTG

TTGTGTCAGGCTCACGGTCTCACTCCAGAACAAGTGGTTGCAATTGCGAGCAATGGA

GGTGGCAAACAAGCTTTGGAGACCGTTCAGCGCTTGCTCCCGGTCTTATGCCAGGCT

CACGGACTTACGCCCCAGCAAGTGGTCGCTATCGCATCCAACGGTGGAGGAAGGCCT

GCACTCGAATCGATTGTGGCACAATTAAGTCGTCCCGACCCTGCCCTTGCAGCGCTG

ACTAATGATCACTTGGTCGCACTCGCGTGCTTAGGTGGAAGACCTGCCCTTGATGCA

GTGAAAAAGGGTCTGCCACATGCTCCCGCACTCATCAAGAGAACTAATAGACGCATA

CCCGAGAGAACCAGCCACCGTGTTGCTGGATCCCAGCTGGTGAAGAGCGAGCTGGAG

GAGAAGAAGTCCGAGCTGCGGCACAAGCTGAAGTACGTGCCCCACGAGTACATCGAG

CTGATCGAGATCGCCAGGAACAGCACCCAGGACCGCATCCTGGAGATGAAGGTGATG

GAGTTCTTCATGAAGGTGTACGGCTACAGGGGAAAGCACCTGGGCGGAAGCAGAAAG

CCTGACGGCGCCATCTATACAGTGGGCAGCCCCATCGATTACGGCGTGATCGTGGAC

ACAAAGGCCTACAGCGGCGGCTACAATCTGCCTATCGGCCAGGCCGACGAGATGGAG

AGATACGTGGAGGAGAACCAGACCCGGAATAAGCACCTCAACCCCAACGAGTGGTGG

AAGGTGTACCCTAGCAGCGTGACCGAGTTCAAGTTCCTGTTCGTGAGCGGCCACTTC

AAGGGCAACTACAAGGCCCAGCTGACCAGGCTGAACCACATCACCAACTGCAATGGC

GCCGTGCTGAGCGTGGAGGAGCTGCTGATCGGCGGCGAGATGATCAAAGCCGGCACC

CTGACACTGGAGGAGGTGCGGCGCAAGTTCAACAACGGCGAGATCAACTTC

>B3-R amino acid sequence

MDYKDHDGDYKDHDIDYKDDDDKMAPKKKRKVGIHGVPMMSRTRLPSPPAPSPAFSA

GSFSDLLRQFDPSLFNTSLFDSLPPFGAHHTEAATGEWDEVQSGLRAADAPPPTMRV

AVTAARPPRAKPAPRRRAAQPSDASPAAQVDLRTLGYSQQQQEKIKPKVRSTVAQHH

EALVGHGFTHAHIVALSQHPAALGTVAVKYQDMIAALPEATHEAIVGVGKQWSGARA

LEALLTVAGELRGPPLQLDTGQLLKIAKRGGVTAVEAVHAWRNALTGAPLNLTPEQV

VAIASHDGGKQALETVQRLLPVLCQAHGLTPQQVVAIASNIGGKQALETVQRLLPVL

CQAHGLTPQQVVAIASNNGGKQALETVQRLLPVLCQAHGLTPEQVVAIASNKGGKQA

LETVQRLLPVLCQAHGLTPEQVVAIASNNGGKQALETVQALLPVLCQAHGLTPEQVV

AIASNGGGKQALETVQALLPVLCQAHGLTPEQVVAIASHDGGKQALETVQALLPVLC

QAHGLTPEQVVAIASNGGGKQALETVQRLLPVLCQAHGLTPEQVVAIASNGGGKQAL

ETVQRLLPVLCQAHGLTPQQVVAIASNNGGKQALETVQRLLPVLCQAHGLTPEQVVA

IASNGGGKQALETVQALLPVLCQAHGLTPEQVVAIASNGGGKQALETVQRLLPVLCQ

AHGLTPEQVVAIASHDGGKQALETVQRLLPVLCQAHGLTPEQVVAIASNIGGKQALE

TVQRLLPVLCQAHGLTPEQVVAIASNNGGKQALETVQRLLPVLCQAHGLTPQQVVAI

ASNKGGRPALETVQRLLPVLCQAHGLTPEQVVAIASNGGGKQALETVQRLLPVLCQA

HGLTPQQVVAIASNGGGRPALESIVAQLSRPDPALAALTNDHLVALACLGGRPALDA

VKKGLPHAPALIKRTNRRIPERTSHRVAGSQLVKSELEEKKSELRHKLKYVPHEYIE

LIEIARNSTQDRILEMKVMEFFMKVYGYRGKHLGGSRKPDGAIYTVGSPIDYGVIVD

TKAYSGGYNLPIGQADEMERYVEENQTRNKHLNPNEWWKVYPSSVTEFKFLFVSGHF

KGNYKAQLTRLNHITNCNGAVLSVEELLIGGEMIKAGTLTLEEVRRKFNNGEI
